# Conversion of Squid Pen to Homogentisic Acid via *Paenibacillus* sp. TKU036 and the Antioxidant and Anti-Inflammatory Activities of Homogentisic Acid

**DOI:** 10.3390/md14100183

**Published:** 2016-10-12

**Authors:** San-Lang Wang, Hsin-Ting Li, Li-Jie Zhang, Zhi-Hu Lin, Yao-Haur Kuo

**Affiliations:** 1 Life Science Development Center, Tamkang University, No. 151, Yingchuan Rd., Tamsui, New Taipei City 25137, Taiwan; 2 Department of Chemistry, Tamkang University, New Taipei City 25137, Taiwan; cindy810924@yahoo.com.tw; 3 Division of Chinese Materia Medica Development, National Research Institute of Chinese Medicine, Taipei 11221, Taiwan; lijiezhang@hotmail.com (L.-J.Z.); tiger77749@gmail.com (Z.-H.L.); 4 Graduate Institute of Integrated Medicine, College of Chinese Medicine, China Medical University, Taichung 40402, Taiwan

**Keywords:** squid pen, chitin, homogentisic acid, tryptophan, *Paenibacillus*, antioxidant, anti-inflammatory

## Abstract

The culture supernatant of *Paenibacillus* sp. TKU036, a bacterium isolated from Taiwanese soils, showed high antioxidant activity (85%) when cultured in a squid pen powder (SPP)-containing medium at 37 °C for three days. Homogentisic acid (2,5-dihydroxyphenylacetic acid, HGA) was isolated and found to be the major antioxidant in the culture supernatant of the SPP-containing medium fermented by *Paenibacillus* sp. TKU036. Tryptophan was also present in the culture supernatant. The results of high-performance liquid chromatography (HPLC) fingerprinting showed that HGA and tryptophan were produced via fermentation but did not pre-exist in the unfermented SPP-containing medium. Neither HGA nor tryptophan was found in the culture supernatants obtained from the fermentation of nutrient broth or other chitinous material, i.e., medium containing shrimp head powder, by *Paenibacillus* sp. TKU036. The production of HGA via microorganisms has rarely been reported. In this study, we found that squid pen was a potential carbon and nitrogen source for *Paenibacillus* sp. Tryptophan (105 mg/L) and HGA (60 mg/L) were recovered from the culture supernatant. The isolated HGA was found to have higher antioxidant activity (IC_50_ = 6.9 μg/mL) than α-tocopherol (IC_50_ = 17.6 μg/mL). The anti-inflammatory activity of the isolated HGA (IC_50_ = 10.14 μg/mL) was lower than that of quercetin (IC_50_ = 1.14 μg/mL). As a result, squid pen, a fishery processing byproduct, is a valuable material for the production of tryptophan and the antioxidant and anti-inflammatory HGA via microbial conversion.

## 1. Introduction

Chitin is one of the most abundant biopolymers in the world, and these natural polymers have versatile properties, such as biocompatibility and non-toxicity. Among the natural chitinous resources, fishery processings (shrimp shells, crab shells, and squid pens) have the highest chitin content. Conventionally, chitin is obtained from shrimp shells, crab shells, and squid pens using a strong alkali or an inorganic acid for deproteinization or demineralization, respectively [1]. However, these chemical processes have several drawbacks, such as the creation of pollutant alkali or acid liquid. Furthermore, the unutilized bioresource of the deproteinized liquid is reduced due to the presence of an alkali [1].

Among the chitin-containing fishery processings, squid pens contain the highest ratio of protein (approximately 70%) [2]. For recycling squid pens in order to produce additional highly value-added products other than chitin or chitosan, we investigated the reutilization of this fishery processings via microbial conversion in order to produce enzymes [2,3,4,5], exopolysaccharides [6,7], chitooligomers [3], antioxidants [8,9], insecticidal materials [10], and biosorbents [11,12].

Many strains of *Paenibacillus* have been reported to use squid pen powder (SPP) as the sole carbon and nitrogen (C/N) source. Recently, we isolated strains of *Paenibacillus* species that converted squid pen to exopolysaccharides [7], chitosanase [3], and chitooligomers [3].

Microbial fermentation can result in the production of some antioxidants, such as ellagic acid produced by *Aspergillus niger* [13], gallic acid produced by *Bacillus sphaericus* [14], ferulic and acid produced by *Saccharomyces cerevisiae* [15]. In this study, we screened antioxidant-producing bacteria from Taiwanese soils by using squid pen as the sole C/N source. A potential bacterial strain, TKU036, was isolated and identified as *Paenibacillus* sp. The optimized culture conditions for antioxidant production via *Paenibacillus* sp. TKU036 was studied.

Here, the antioxidant compound produced in the culture supernatant of *Paenibacillus* sp. TKU036 was isolated and identified as HGA. HGA has shown to have antioxidant and anti-inflammatory activities [16,17]. In this study, the antioxidant and anti-inflammatory activities of the isolated HGA were investigated and compared with those activities of other well-known antioxidant (α–tocopherol) and anti-inflammatory compound (quercetin).

## 2. Results and Discussion

### 2.1. Screening and Identification of Strain TKU036

Over 350 bacterial strains isolated from the soils of Northern Taiwan were cultivated at 37 °C in a medium containing 1% squid pen powder (SPP). Among these strains, strain TKU036 exhibited the strongest antioxidant activity and was chosen for more intensive examination. Based on morphological and biochemical studies, as well as 16S rDNA sequences [7], this strain was confirmed to be *Paenibacillus* sp. Analytical profile index (API) identification was further used to identify the species name [7]; however, no match was found. Therefore, the TKU036 strain was identified as *Paenibacillus* sp. and was used for further investigation.

### 2.2. Comparing the Non-Exopolysaccharide Antioxidants Produced by Paenibacillus Species

Many strains of *Paenibacillus*, such as *P. mucilaginosus* TKU032 [7], *Paenibacillus* sp. TKU023 [18], and *P. macerans* TKU029 [19], have been reported as the sole C/N source for the production of exopolysaccharides (EPOs) using SPP, and some of these EPOs showed antioxidant activity. In this study, EPOs were also found in the culture broth of SPP-containing medium fermented by *Paenibacillus* sp. TKU036 (data not shown). To investigate whether the antioxidant activity was from the EPOs, the EPO-containing culture supernatant of strain TKU036 underwent ethanol precipitation (final concentration of 70%, *v*/*v*) to remove the EPOs. The obtained EPO-deficient culture supernatants were than lyophilized to remove the ethanol and were used for analyzing antioxidant activity. The EPO-deficient culture supernatant of *Paenibacillus* sp. TKU036 showed high antioxidant activity (85%). The culture conditions for the production of antioxidants and the isolation of the non-EPO antioxidants were studied subsequently.

### 2.3. Culture Conditions for Antioxidant Production

Different concentrations (0.5%, 1.0%, and 1.5% *w*/*v*) of squid pen powder (SPP), shrimp head powder (SHP), and cicada shell powder (CSP) were used as the sole C/N source for the production of antioxidant by *Paenibacillus* sp. TKU036. The effects of the medium volume, the medium pH, and the culture temperature on antioxidant activity were also examined. Commercial nutrient broth (NB) medium, which does not contained chitin, was used for comparison. The result showed that the highest antioxidant activity (85%) was obtained by fermentation within the 0.5% SPP-containing medium (100 mL medium in 250 mL Erlenmeyer flask) at 37 °C in a reciprocal shaker at 150 rpm for three days.

The results of this study are remarkably different from those of other reports, such as studies of Bacillus subtilis using red bean [20], *Aspergillus awamori* and *Aspergillus oryzae* using soybean [21], *Aspergillus usami* using sesamin [22], *A. awamori* using black bean [23], and *Monascus pilosus* using potato dextrose broth [24] as a C/N source for antioxidant production.

A novel antioxidant (serraticin) with antitumor activity was isolated from the culture supernatant of SPP-containing medium fermented by *Serratia ureilytica* TKU013 [8]. *S. ureilytica* TKU013 used SPP (1.5%) for the production of the antioxidant, but the maximal antioxidant activity was 82% after four days of fermentation [25]. In this study, *Paenibacillus* sp. TKU036 used a cheaper C/N source of 0.5% SPP and produced a higher antioxidant activity (85%) in a shorter time (three days). The studied culture condition was then used for antioxidant production.

### 2.4. Isolation of Antioxidant Compounds

**Figure 1:**
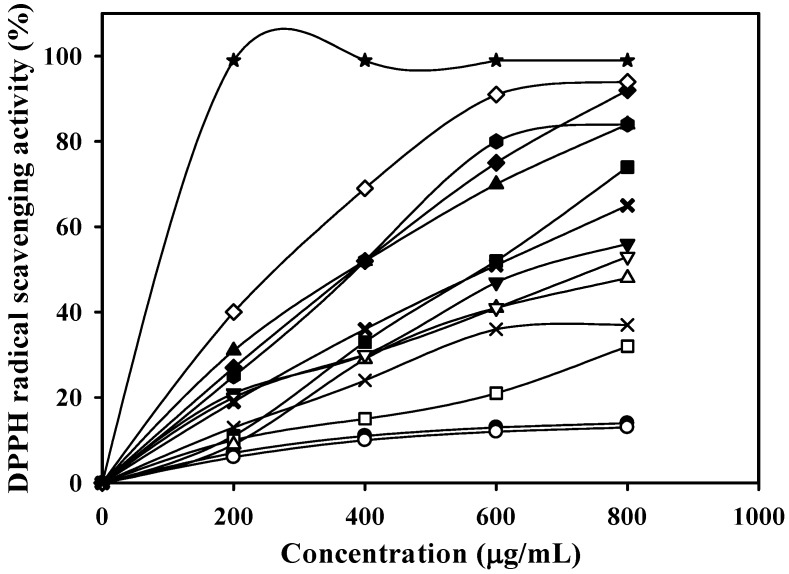
DPPH radical scavenging activity of the 14 fractions eluted by different concentrations of methanol. —●—, Fraction 1 (1.1414 g, eluted with 0% methanol); —○—, Fraction 2 (0.4464 g, eluted with 5% methanol); —
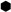
—, Fraction 3 (0.1765 g, eluted with 10% methanol); —★—, Fraction 4 (0.1698 g, eluted with 15% methanol); —■—, Fraction 5 (0.1253 g, eluted with 20% methanol); —□—, Fraction 6 (0.0864 g, eluted with 25% methanol); —◆—, Fraction 7 (0.0599 g, eluted with 30% methanol) ;—◇—, Fraction 8 (0.0482 g, eluted with 35% methanol); —▲—, Fraction 9 (0.0363 g, eluted with 40% methanol); —△—, Fraction 10 (0.0275 g, eluted with 45% methanol); —▼—, Fraction 11 (0.0263 g, eluted with 50% methanol); —▽—, Fraction 12 (0.0321 g, eluted with 55% methanol); —✖—, Fraction 13 (0.0324 g, eluted with 60% methanol); and —✕—, Fraction 14 (0.0424 g, eluted with 100% methanol).

As described in the Materials and Methods section below, the 95% ethanol extract was separated into 14 fractions by column chromatography. All the fractions were evaluated for antioxidant activity using a scavenging 2,2-diphenyl-1-picrylhydrazyl (DPPH) radical test. As shown in Figure 1, Fraction 4 had the highest antioxidant activity.

**Figure 2:**
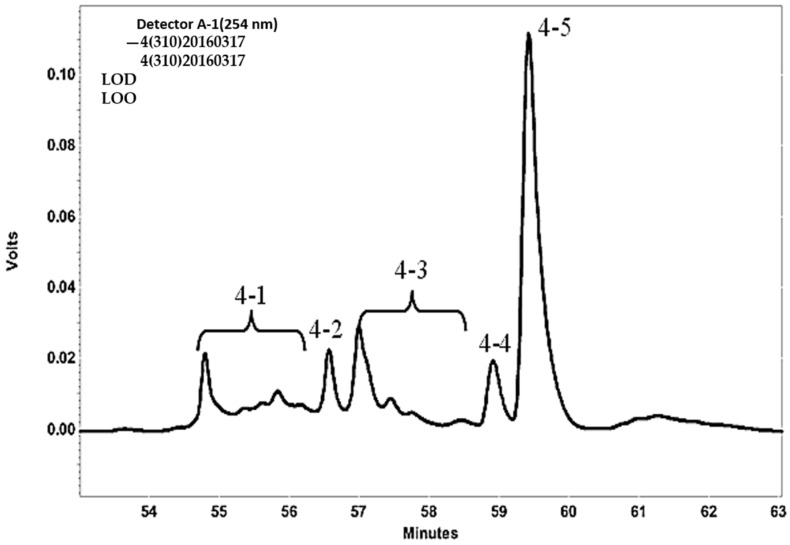
The HPLC profile of Fraction 4 (13% acetonitrile, 254 nm).

**Figure 3:**
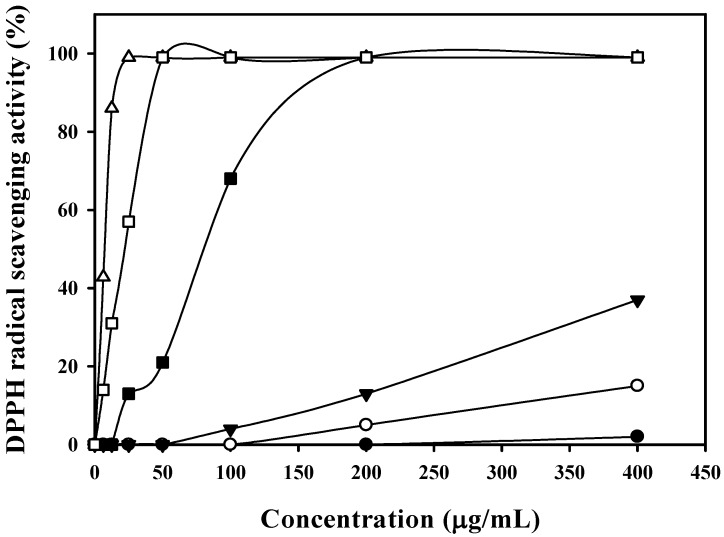
DPPH radical scavenging activities of 4-1 to 4-5. —●—, 4-1; —○—, 4-2; —▼—, 4-3; —△—, 4-4; —■—, 4-5; and —□—, α-tocopherol.

At a concentration of 200 μg/mL, Fraction 4 showed approximately 99% antioxidant activity. Fraction 4 was further purified with a preparative HPLC column. In total, five sub-fractions (4-1, 4-2, 4-3, 4-4, and 4-5) (Figure 2) were obtained, and the antioxidant activity of these fractions were analyzed (Figure 3). As shown in Figure 3, Fraction 4-4 showed the highest antioxidant activity (IC_50_ of 6.9 μg/mL) compared with those of Fraction 4-5 (62.8 μg/mL, which showed a little antioxidant activity due to containing minor 4-4) and the other three fractions, as well as the antioxidant activity of the positive control, α-tocopherol (17.6 μg/mL). The results showed Fraction 4-4 contained a potential antioxidant that was valuable for further identification.

### 2.5. Identification of HGA and Tryptophan by NMR

**Figure 4:**
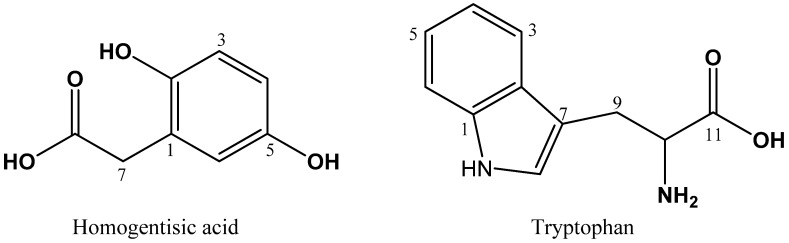
The chemical structures of HGA (**left**) and l-tryptophan (**right**).

The chemical structures of the isolated compounds were elucidated using detailed spectroscopic analyses, including 1D (^1^H NMR, ^13^C NMR) and 2D NMR experiments (^1^H–^1^H COSY, HSQC, and HMBC), together with the spectroscopic comparisons of previously reported compounds. Fractions 4-4 and 4-5 were shown to contain HGA [26] and tryptophan [27], respectively (Figure 4).

HGA (4-4) was obtained as a white amorphous powder. ^1^H NMR data (400 MHz, MeOH-*d_4_*, *δ*_H_ ppm): 6.62 (d, *J* = 8.4 Hz, H-3), 6.59 (d, *J* = 2.8 Hz, H-6), 6.53 (dd, *J* = 8.4, 2.8 Hz, H-4), and 3.50 (s, 2H, H-7). ^13^C NMR data (100 MHz, MeOH-*d_4_*, *δ*_C_ ppm): 177.0 (C-8), 151.6 (C-5), 150.4 (C-2), 124.3 (C-1), 119.0 (C-6), 117.4 (C-3), 116.0 (C-4), and 37.7 (C-7).

Tryptophan was obtained as a white amorphous powder. ^1^H NMR data (600 MHz, MeOH-*d_4_*, *δ*_H_ ppm): 7.69 (d, *J* = 7.8 Hz, H-3), 7.35 (d, *J* = 7.8 Hz, H-6), 7.19 (s, H-8), 7.11 (td, *J* = 7.8, 0.6 Hz, H-5), 7.04 (td, *J* = 7.8, 0.6 Hz, H-4), 3.86 (dd, *J* = 9.6, 4.2 Hz, H-10), 3.51 (dd, *J* = 15.6, 9.6 Hz, H-9), 3.15 (dd, *J* = 15.6, 4.2 Hz, H-9). ^13^C NMR data (150 MHz, MeOH-*d_4_*, *δ*_C_ ppm): 174.5 (C-11), 138.4 (C-1), 128.5 (C-2), 125.2 (C-8), 122.7 (C-5), 120.1 (C-4), 119.3 (C-3), 112.4 (C-6), 109.5 (C-7), 56.7 (C-10), and 28.5 (C-9).

HGA is an important intermediate in the metabolism of phenylalanine and tyrosine [28]. The production HGA via microorganisms has only been shown in a few reports, such as *Aspergillus niger* (using phenyl acetic acid as a C/N source) [29], *Vibrio cholerae* (using marine broth with 4 mM tyrosine as a C/N source) [30], and *Yarrowia lipolytica* (using tyrosine as a C/N source) [28]. Tryptophan is widely used in human food and medicine, as well as in animal feed. The production of tryptophan has been reported in two typical bacteria strains: *Escherichia coli* FB-04 (using glucose, yeast, tryptone, and citric acid as C/N sources) [31] and *Corynebacterium glutamicum* KY9218 (using sucrose, corn steep liquor, tyrosine, phenylalanine etc. as C/N sources) [32]. In this study, *Paenibacillus* sp. TKU036 cultured with SPP, a seafood processing, was used for the production of HGA and tryptophan and may have potential for further investigation.

### 2.6. The Effect of HGA on Cytotoxicity and Anti-Inflammation

**Figure 5:**
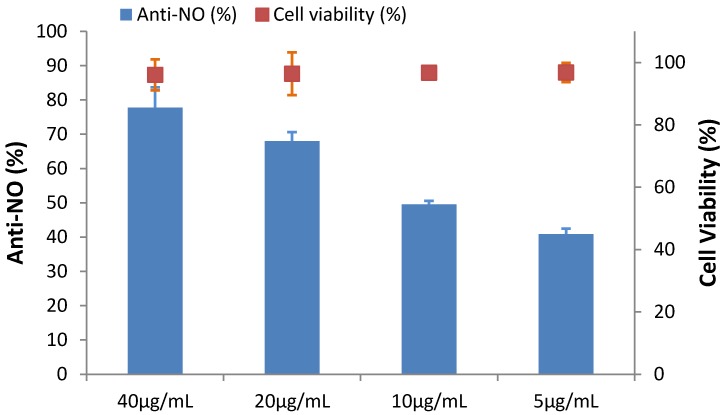
NO inhibitory activities of HGA isolated from culture supernatant of *Paenibacillus* sp. TKU036 in the SPP-containing medium. Cell lines: Murine RAW 264.7 monocyte/macrophage cells. The cells were treated with LPS (1 μg/mL) or in combination with the tested agents (40, 20, 10, and 5 μg/mL) for 24 h.

Nitric oxide (NO) is recognized as a key pro-inflammatory mediator that is involved in certain inflammatory disorders, including chronic hepatitis, pulmonary fibrosis, and rheumatoid arthritis [33]. In our previous study [3], we discovered chitosan oligomers with a low degree of polymerization that showed both antioxidant and anti-inflammatory activity. In this study, the anti-inflammatory activity of Fraction 4-4 (HGA) was estimated using an in vitro model: LPS-stimulated RAW 264.7 cells. The inhibition of LPS-stimulated NO secretion was due to anti-inflammatory activity. First, to examine the potential cell cytotoxicity induced by Fraction 4-4, a MTT assay was conducted. When RAW 264.7 macrophages were treated with Fraction 4-4 at concentrations of 0, 5, 10, 20, and 40 μg/mL, along with 1 μg/mL LPS, the resulting viabilities of RAW 264.7 cells were recorded and are summarized in Figure 5. The results of a statistical analysis indicated that treatment with Fraction 4-4 (40 μg/mL) had no noticeable toxic effect on cell growth when compared with the cell growth of the 0.05% DMSO treated group (100%). Fraction 4-4 (40 μg/mL) was capable of inhibiting NO production by 77.79% in LPS-stimulated cells. The IC_50_ value of Fraction 4-4, representing the anti-inflammatory effect, was 10.14 μg/mL (Figure 5). A similar result was found when purchased HGA was used. Quercetin is a potent dietary antioxidant that also displays anti-inflammatory activity [34]. Thus, the anti-inflammatory activity (IC_50_) of quercetin was investigated. These results indicated that HGA exhibited an acceptable anti-inflammatory activity (IC_50_ = 10.14 μg/mL) compared with the anti-inflammatory activity of quercetin (IC_50_ = 1.14 μg/mL).

### 2.7. Confirmation of HGA and Tryptophan Produced from SPP by Fermentation

The 95% ethanol extracts were extracted from the culture fermented supernatant of *Paenibacillus* sp. TKU036 and were then compared with the extract of unfermented medium via HPLC analysis. The results found that HGA and tryptophan appeared in the fermentation supernatant at *R*t 12.75 min and at *R*t 16.25 min, respectively, revealing that the two components did not pre-exist in the SPP-containing medium (data not shown).

**Figure 6:**
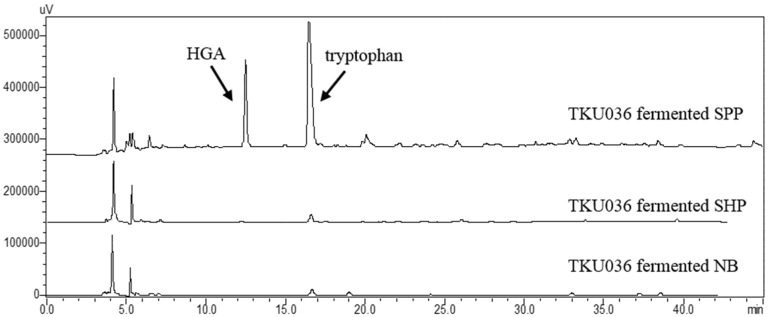
The HPLC fingerprints of the ethanol extracts from the *Paenibacillus* sp. TKU036 fermented SPP-containing medium, the *Paenibacillus* sp. TKU036 fermented SHP-containing medium, and *Paenibacillus* sp. TKU036 fermented nutrient broth (NB).

To confirm whether HGA and tryptophan were also produced using other chitin-containing materials as the sole C/N source, the 0.5% SHP-containing medium was also studied. Furthermore, nutrient broth (NB), a commercial medium for bacteria cultivation, was also tested. As shown in Figure 6, after fermentation by *Paenibacillus* sp. TKU036 for three days, no HGA and tryptophan were detected from the ethanol extract of the culture supernatant. To the best of our knowledge, there have been no reports of materials harmful to humans produced by *Paenibacillus* species [35]. The transformation of squid pen to functional foods of HGA and tryptophan via *Paenibacillus* sp. strain TKU036 may have the potential to be intensively investigated.

## 3. Materials and Methods

### 3.1. Materials

Squid pens were obtained from Shin-Ma Frozen Food Co. (I-Lan, Taiwan). Shrimp head power (SHP) was obtained from Fwu-Sow Industry. (Taichun, Taiwan). Cicada shells were collected at the Tamsui Campus of Tamkang University (New Taipei, Taiwan). HGA, tryptophan, and 2,2-diphenyl-1-picrylhydrazyl (DPPH) were purchased from Sigma-Aldrich (St. Louis, MO, USA). Nutrient broth was obtained from Difco. Octadecylsilane (ODS) gel was purchased from Merck (Darmstadt, Germany).

### 3.2. Antioxidant Activity Assay

The antioxidant samples (1.2 mL) were mixed with 0.3 mL of a methanolic solution containing 0.75 mM DPPH radicals. The mixture was vigorously shaken and incubated for 30 min in the dark, and the absorbance was then measured at 517 nm against a blank [3]. The scavenging ability was calculated as described in our previous paper [3].

### 3.3. Screening of Antioxidant-Producing Strain

The bacteria were isolated from soil samples that were collected at different locations in Northern Taiwan. They were cultivated in a medium containing squid pen powder (SPP) (pH 7.2) supplemented with 0.05% MgSO_4_·7H_2_O and 0.1% K_2_HPO_4_ to screen for antioxidant activity. The strains were cultivated in a 250 mL Erlenmeyer flask that contained 50 mL of medium at 37 °C in a reciprocal shaker at 150 rpm for 1–2 days. The supernatants obtained via centrifugation were used for the estimation of antioxidant activity using the protocol described in our previous paper [7]. Strain TKU036, which showed the highest activity, was selected for further study.

### 3.4. Extraction and Isolation of HGA and Tryptophan

The culture supernatant (2 L) of *Paenibacillus* sp. TKU036 was lyophilized (5.824 g) and extracted via ultrasonication in 300 mL of 95% ethanol at 60 °C in triplicate. The extract was concentrated under reduced pressure. The obtained ethanol extract (2.4905 g) was dissolved in H_2_O, and it was loaded onto an open ODS column and eluted with 0%–60% MeOH in H_2_O (to maintain the acetic acid concentration at 0.4%), resulting in 14 fractions (Fractions 1 to 14). The tryptophan was found in Fractions 5 (0.1253 g) and 6 (0.0864 g). Fraction 4 was further purified via preparative HPLC, equipped with a 250 mm × 20 mm i.d. preparative Cosmosil 5C18-AR-II column (Nacalai Tesque, Kyoto, Japan) and a UV detector at 254 nm, and it was eluted with 13% ACN in H_2_O (0.4% acetic acid), yielding 5 fractions (Fractions 4-1 to 4-5). HGA (0.012 g) and tryptophan (0.008 g) were obtained from Fractions 4-4 and 4-5, respectively. In total, HGA (0.012 g) and tryptophan (0.21 g) were produced from 2 L of the culture supernatant (5.824 g) by employing the method described above. The recovery of HGA (60 mg/L) in this study was comparable to that of HGA in strawberry tree honey, which has been reported to contain HGA at a concentration of 414 mg/kL [19].

### 3.5. The Analysis of HGA and Tryptophan by HPLC:

The chromatographic separation was carried out on an Agilent HC-C18 (5 μm, 250 mm × 4.6 mm i.d., Agilent Technologies, Tokyo, Japan). The binary gradient elution system consisted of 0.4% acetic acid aq. (A) and 0.4% acetic acid in acetonitrile (B), and the HPLC profile separation was achieved using the following gradient: 0–20 min, 5%–15% B; 20–35 min, 15%–35% B; and 35–40 min, 35%–100% B. The UV detection wavelength was 292 nm. The column was kept at room temperature. The flow rate was 0.8 mL/min, and the injection volume was 10 μL.

## 4. Conclusions

To efficiently reutilize seafood processings via microbial transformation, squid pen was used as the sole C/N source for screening antioxidant-producing bacteria from Taiwanese soils. The culture supernatant of strain TKU036 produced potential antioxidant activity and was identified as *Paenibacillus* species. The antioxidant compound in the culture supernatant was identified as HGA, which showed anti-inflammatory effects as well. Tryptophan was also identified in the culture supernatant. Neither HGA nor tryptophan was found in the unfermented SPP-containing medium or in other chitinous materials (shrimp head powder)-containing medium. The results showed that squid pen is a promising material for the production of antioxidants and anti-inflammatories by *Paenibacillus* sp. TKU036.

HGA showed higher antioxidant activity (IC_50_ = 6.9 μg/mL) than α-tocopherol (IC_50_ = 17.6 μg/mL). The anti-inflammation activity of HGA (IC_50_ = 10.14 μg/mL) was lower than that of quercetin (IC_50_ = 1.14 μg/mL). HGA has been reported as the most abundant phenolic compound in strawberry tree honey (414 mg/kg) [36]. The recovery of 60 mg of HGA per liter of culture supernatant, compared with that of strawberry tree honey, seems to have potential for HGA production.
